# Artificial Intelligence in Scoliosis Classification: An Investigation of Language-Based Models

**DOI:** 10.3390/jpm13121695

**Published:** 2023-12-09

**Authors:** Artur Fabijan, Bartosz Polis, Robert Fabijan, Krzysztof Zakrzewski, Emilia Nowosławska, Agnieszka Zawadzka-Fabijan

**Affiliations:** 1 Department of Neurosurgery, Polish-Mother’s Memorial Hospital Research Institute, 93-338 Lodz, Poland; jezza@post.pl (B.P.); krzysztof.zakrzewski@iczmp.edu.pl (K.Z.); emilia.nowoslawska@iczmp.edu.pl (E.N.); 2 Independent Researcher, Luton LU2 0GS, UK; robert.f.fabijan@gmail.com; 3 Department of Rehabilitation Medicine, Faculty of Health Sciences, Medical University of Lodz, 90-419 Lodz, Poland; agnieszka.zawadzka@umed.lodz.pl

**Keywords:** artificial intelligence, scoliosis, ChatGPT, Microsoft Bing, Scholar AI

## Abstract

Open-source artificial intelligence models are finding free application in various industries, including computer science and medicine. Their clinical potential, especially in assisting diagnosis and therapy, is the subject of increasingly intensive research. Due to the growing interest in AI for diagnostics, we conducted a study evaluating the abilities of AI models, including ChatGPT, Microsoft Bing, and Scholar AI, in classifying single-curve scoliosis based on radiological descriptions. Fifty-six posturographic images depicting single-curve scoliosis were selected and assessed by two independent neurosurgery specialists, who classified them as mild, moderate, or severe based on Cobb angles. Subsequently, descriptions were developed that accurately characterized the degree of spinal deformation, based on the measured values of Cobb angles. These descriptions were then provided to AI language models to assess their proficiency in diagnosing spinal pathologies. The artificial intelligence models conducted classification using the provided data. Our study also focused on identifying specific sources of information and criteria applied in their decision-making algorithms, aiming for a deeper understanding of the determinants influencing AI decision processes in scoliosis classification. The classification quality of the predictions was evaluated using performance evaluation metrics such as sensitivity, specificity, positive predictive value (PPV), negative predictive value (NPV), accuracy, and balanced accuracy. Our study strongly supported our hypothesis, showing that among four AI models, ChatGPT 4 and Scholar AI Premium excelled in classifying single-curve scoliosis with perfect sensitivity and specificity. These models demonstrated unmatched rater concordance and excellent performance metrics. In comparing real and AI-generated scoliosis classifications, they showed impeccable precision in all posturographic images, indicating total accuracy (1.0, MAE = 0.0) and remarkable inter-rater agreement, with a perfect Fleiss’ Kappa score. This was consistent across scoliosis cases with a Cobb’s angle range of 11–92 degrees. Despite high accuracy in classification, each model used an incorrect angular range for the mild stage of scoliosis. Our findings highlight the immense potential of AI in analyzing medical data sets. However, the diversity in competencies of AI models indicates the need for their further development to more effectively meet specific needs in clinical practice.

## 1. Introduction

Artificial Intelligence (AI) is a field of science and technology that mimics human cognitive abilities, enabling machines to learn and make decisions. In recent years, AI has revolutionized many aspects of daily life, including medicine, where it is increasingly used in diagnostics and treatment planning [1,2,3,4].

Open-source artificial intelligence models (OSAIM) are typically available for free and can be publicly utilized, offering support in diverse sectors, including fields such as computer science and medicine [5,6]. Applications belonging to the OSAIM category are being increasingly explored for their clinical applications, particularly as tools aiding medical diagnostics and the selection of appropriate therapeutic treatments.

The Generative Pre-trained Transformer (GPT), developed by OpenAI, represents the pinnacle of current achievements in language models, employing advanced machine learning techniques, particularly deep learning methods [7]. This model exhibits exceptional competence in generating texts characterized by coherence and relevance, enabling its application in a broad spectrum of fields, ranging from automated text production and machine language translation to chatbot implementation [8]. This model is also capable of assimilating knowledge from various fields, making it an increasingly researched tool in scientific communities such as biology and medicine [9,10].

Over time, artificial intelligence evolves, expanding its capabilities through additional tools such as Microsoft Bing and Scholar AI. Bing introduces advanced search and text analysis algorithms that allow for a deeper understanding and interpretation of data [11]. Meanwhile, Scholar AI utilizes natural language processing and machine learning technologies to conduct a precise analysis of large scientific datasets, enabling more accurate inference and identification of trends in research [12]. Scholar AI is a model designed to provide users access to a database of peer-reviewed articles and scientific research. It combines natural language models (LLMs) used by ChatGPT with tailored access to open scientific articles. Users can directly search for relevant peer-reviewed works to obtain reliable information necessary for their scientific research, technical projects, and funding proposals. Scholar AI is available in three versions: free, basic, and premium. The free version allows access to basic functions, such as browsing peer-reviewed scientific publications with citations and source materials. The basic version offers unlimited access to an advanced literature search tool with over 40 million articles from PubMed, Springer, Arxiv, Science, IEEE, and more, as well as a customized summary function tailored to academic articles. The premium version provides unrestricted access to the latest features, including everything offered in the basic version, plus the ability to ask questions directly of academic PDFs and extract figures and tables from PDF documents, which is currently in beta [12]. 

Microsoft Bing, utilizing advanced ChatGPT technologies, presents itself as an innovative online assistant. Its primary function is efficient web searching to provide users with the most relevant and precise information, significantly reducing search time. A key feature of Bing Chat is its adaptability in dialogue style, allowing users to personalize interactions. This assistant offers a wide range of possibilities: from searching for facts and information, through creating creative content, to conducting casual conversations tailored to user needs and preferences [13].

The integration of AI in the medical field, particularly in neurosurgery and spine health, has been a significant advancement. Recent studies [14] highlight the transformative impact of AI in these areas. AI’s application in neurosurgery encompasses a range of activities from diagnostic procedures to surgical planning and execution, offering unprecedented precision and efficiency. This is particularly relevant in the context of spinal health, where AI’s analytical capabilities can play a crucial role in diagnosing and treating complex conditions.

**Figure 1:**
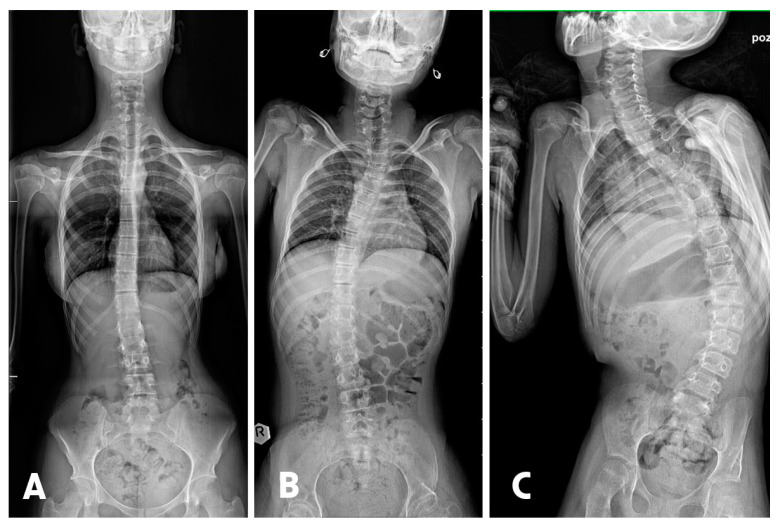
(**A**) presents a mild form of scoliosis, where the Cobb angle is 12 degrees, measured between the L1/L2 and Th8/Th9 vertebrae. (**B**) shows moderate scoliosis with a curvature angle of 32 degrees, measured between the L1/L2 and Th6/Th7 vertebrae. (**C**) represents severe single-curve scoliosis, with a Cobb angle reaching 56 degrees, measured between the L3/L4 and Th6/Th7 vertebrae.

Scoliosis is a condition characterized by lateral curvature of the spine, with a Cobb angle in the frontal plane exceeding 10 degrees [15]. Scoliosis can be categorized into several groups based on various criteria, such as cause, location, curvature pattern, age of onset, and severity. The most fundamental divisions include curvature patterns such as C-Shaped: single curve or S-Shaped: double curve and according to the severity of the curvature based on Cobb angle measurements. When the Cobb angle does not exceed 10 degrees, we observe a minor spinal deviation, not classified as scoliosis. Diagnoses with Cobb angles between 10 and 20 degrees indicate a mild form of scoliosis. Moderate scoliosis is characterized by a Cobb angle of 20 to 40 degrees. When this angle exceeds 40 degrees, we are dealing with advanced, severe scoliosis [16,17] (Figure 1).

In light of continuous advancements in AI, its applications in assessing conditions such as scoliosis open new diagnostic perspectives. Traditionally, most AI models used in this medical sphere are precisely trained using radiological images, enabling accurate identification and classification of spinal deformity [18]. However, there has been a growing interest in the potential and usefulness of models based on natural language processing, such as ChatGPT and Microsoft Bing, which, instead of image analysis, utilize clinical textual data to generate diagnoses and recommendations [19,20]. 

Our previous study sheds light on the capabilities of the publicly accessible contrastive language–image pretraining (CLIP) system in recognizing severe cases of single-curve scoliosis. The results showed that although some AI models possess the ability to partially recognize scoliosis based on visual data, their effectiveness is limited [21]. Consequently, in our article, we delve into the current state of research on the application of AI models in medicine, highlighting that they are rarely considered in the context of scoliosis, despite the potential to offer new, valuable diagnostic perspectives.

In response to the growing interest in the use of AI as a diagnostic tool, a study was conducted aimed at evaluating the accuracy and interpretative capabilities of AI models such as ChatGPT, Microsoft Bing, and Scholar AI in the classification of single-curve scoliosis as mild, moderate, or severe. The study compares these models amongst themselves as well as with traditional diagnostic methods used by specialists, taking into account the scientific sources upon which these models rely for classification. 

### Research Hypothesis

In our study, we decided to verify that all selected AI models will correctly classify cases of single-curve scoliosis, based on the Cobb angle measurement method. We anticipate that these results will contribute to the development of knowledge regarding the use of artificial intelligence in medicine, particularly in diagnosing and monitoring the progression of scoliosis.

## 2. Materials and Methods

This study was conducted as part of the scientific research activities at the Polish-Mother’s Memorial Hospital Research Institute in Poland. Radiological posturographic images in the anteroposterior (AP) projection, diagnosing cases of scoliosis, were utilized. The research period spanned from February 2023 to October 2023, and the subjects represented an age group ranging from 2 to 17 years. Out of 137 collected study results, 56 cases of single-curve scoliosis were qualified for further analysis. Radiological diagnostic examinations are conducted solely based on established clinical indications. Therefore, this study only analyzed radiological images of patients with previously diagnosed scoliosis. All personal information related to the subjects was anonymized. The study was conducted in accordance with the guidelines of the ethics committee, which confirmed that no additional consent was required.

All radiological examinations included in this study were performed using standardized, high-precision equipment, specifically focusing on cases of single-curve scoliosis. The criteria for inclusion required that each image be technically correct, clear, and free from artifacts, displacements, or errors in film splicing. Cases of other types of scoliosis, images of poor quality or damaged, as well as those not showing the entire spine or having visible stabilizing systems, were excluded.

Two independent neurosurgery specialists (B.P.; E.N.) analyzed the selected set of 56 posturographic AP projection images with visible single-curve scoliosis. This scoliosis was characterized by a diversity in curvature angles, ranging from 11 to 96 degrees. 

The images were used only to develop basic descriptions of scoliosis cases and were not exported to external servers. The created radiological descriptions of scoliosis included key parameters: the degree of deformation measured by the Cobb method, precise identification of the spine segment affected by the deformation, and determination of whether the deformation is right-sided or left-sided. These descriptions were then used to evaluate the potential and accuracy of classification by open AI systems.

### 2.1. Manual Measurement

Analysis of the posturographic X-ray images was conducted independently by two neurosurgery specialists. RadiAnt (version 2023.1) software was used to evaluate the posturographic images and the Cobb angle measurements.

### 2.2. AI Systems Evaluation Methodology

The study utilized three open-source code-based programs: ChatGPT 4, Microsoft Bing, and Scholar AI (in both free and Premium versions). Each system was tested separately, using the same question and previously developed descriptions of scoliosis. The models were tested on 29 October 2023.

First command: ‘Based on the following radiological descriptions, classify the scoliosis based on Cobb angles’.

Subsequently, the 56 developed descriptions of single-curve scoliosis were entered simultaneously.

After receiving the responses, each model was asked to justify, based on what data, the scoliosis classification.

Second command: ‘What source data did you base the above classification on?’.

### 2.3. Data Analysis

The assessment was conducted by two independent neurosurgery specialists utilizing Cobb’s angle method. The classification criteria were as follows: curvatures ranging between 10 and 20 degrees (inclusive) were categorized as mild scoliosis; angles between 20 and 40 degrees (inclusive) were deemed moderate; and any curvature surpassing 40 degrees was classified as severe scoliosis. For the purpose of the analysis, only the measurement results demonstrating complete interrater reliability between specialists were selected. These results were then treated as the actual outcomes. 

The classification quality of the predictions was evaluated utilizing performance evaluation metrics such as sensitivity, specificity, positive predictive value (PPV), negative predictive value (NPV), accuracy, and balanced accuracy, as defined by Equations (1) through (6) [22,23,24]. These metrics are typically pertinent to binary classification scenarios; however, in the context of a multi-class classification task, these metrics were computed for each class against the aggregate of the remaining classes. Thus, this modified approach allowed for a nuanced, class-specific assessment of the model’s predictive performance. The global accuracy rate was calculated, accompanied by a 95% confidence interval (CI 95%) for this rate, employing the binomial test.

(1) $${Sensitivity}_{i}=\frac{{TP}_{i}}{{TP}_{i}+{FN}_{i}}$$

(2) $${Specificity}_{i}=\frac{{TN}_{i}}{{TN}_{i}+{FP}_{i}}\;$$

(3) $${PPV}_{i}=\frac{{TP}_{i}}{{TP}_{i}+{FP}_{i}}\;$$

(4) $${NPV}_{i}=\frac{{TN}_{i}}{{TN}_{i}+{FN}_{i}}$$

(5) $${Accuracy}_{i}=\frac{{TP}_{i}+{TN}_{i}}{{TP}_{i}+{TN}_{i}+{FP}_{i}+{FN}_{i}}\;$$

(6) $${Balanced\;accuracy}_{i}=\frac{{Sensitivity}_{i}+{Specificity}_{i}}{2}\;$$

where *TP_i_*—a True Positives for class *i*; *TN_i_*—True Negatives for class *i*; *FP_i_*—False Positives for class *i*; *FN_i_*—False Negatives for class *i.*

The interrater agreement between actual and predicted data was estimated by Fleiss’ Kappa [25].

The average of the absolute difference between the predicted and actual values was assessed by Mean Absolute Error (MAE) by Equation (7). Such a measure allowed estimating the distance between the current size of the Cobbs angle and the predicted category.

(7) $$MAE=\frac{1}{N}\cdot \sum \left|y_{pred\;i}-y_{actual\;i}\;\right|$$

where *i* = 1, …, *N*.

In the scenario where the class of scoliosis was accurately predicted, the absolute difference between the predicted value $y_{pred\;i}$ and the actual value $y_{actual\;i}$ equaled zero. This indicated a perfect match between the predicted and actual class. Conversely, in instances of inaccurate classification, the absolute difference $\left|y_{pred\;i}-y_{actual\;i}\right|$ signified the distance to the closest score of the correctly classified scoliosis class. This distance provided a quantifiable measure of the discrepancy between the model’s prediction and the actual classification, thereby serving as an indicator of the prediction error.

### 2.4. Statistical Environment

The analyses were carried out using the statistical language R (version 4.3.1) [26] on Windows 10 Pro 64 bit (build 19045), using the packages lpSolve (version 5.6.19) [27], irr (version 0.84.1) [28], caret (version 6.0.94) [29], report (version 0.5.7) [30], ggplot2 (version 3.4.4) [31], and psych (version 2.3.9) [32].

## 3. Results

### 3.1. Characteristics of the Sample

The dataset under consideration encompassed Cobb’s angle values extracted from 56 posturographic images captured in the anteroposterior (AP) projection. These images exhibited the presence of apparent single-curve scoliosis. An examination of the descriptive statistics revealed that these angles possessed a mean value of *M* = 37.64 degrees, accompanied by a standard deviation of *SD* = 25.07 degrees. The scope of Cobb’s angles in the dataset was fairly broad, with the smallest and largest values being *Min* = 11 degrees and *Max* = 92 degrees, respectively, resulting in a range of 81 degrees. The central 50% of values in the dataset lied within the 15.75–56.76 range.

The distribution of scoliosis severity, as evaluated by neurosurgery specialists (actual values), exhibited the following pattern: a total of *n*_1_ = 22 cases (representing 39.3% of the findings) were identified as mild scoliosis. In contrast, *n*_2_ = 10 instances (17.9%) fell under the category of moderate scoliosis. The majority of the findings, *n*_3_ = 24 cases (42.8%), were classified as severe scoliosis. This distribution highlights the skew towards more severe manifestations of scoliosis within the evaluated dataset.

#### Comparison of Actual and AI-Predicted Scoliosis Classifications: An Analysis of Performance Evaluation Metrics

From Table 1, it can be concluded that the ChatGPT 4 and Scholar AI Premium models demonstrated superior performance across all scoliosis severity classes, achieving perfect interrater agreement and performance evaluation metrics. In contrast, the Microsoft Bing and Scholar AI Free models showed high agreement levels and predictive metrics, although struggled somewhat with accurately classifying moderate and severe cases.

### 3.2. Fleiss Kappa 

Both models demonstrated high levels of interrater agreement for the mild and severe scoliosis classes, indicating their reliability in these classifications. However, the Scholar AI Free model showed superior performance, achieving perfect agreement in the severe class and marginally higher overall agreement across all cases.

### 3.3. Sensitivity

The models showed high sensitivity in predicting the severe class, with Scholar AI Free demonstrating perfect sensitivity for moderate cases. This indicated that Scholar AI Free is slightly more reliable in correctly identifying positive cases.

### 3.4. Specificity

Both models performed perfectly for the mild class, but Scholar AI Free outperformed Microsoft Bing for the moderate and severe classes. This suggested that Scholar AI Free is more effective in correctly identifying negative cases across all severity classes.

### 3.5. Positive Predictive Value

Both the Microsoft Bing and Scholar AI Free models perfectly predicted mild scoliosis cases. However, for moderate and severe classes, their performances varied. Microsoft Bing had higher precision in moderate cases, while Scholar AI Free exhibited perfect precision for severe cases.

### 3.6. Negative Predictive Value

For the mild and moderate scoliosis classes, there were notable differences between the models. The Microsoft Bing model was more effective in predicting non-mild cases, while Scholar AI Free demonstrated a perfect NPV for non-moderate cases, indicating its superior ability to avoid false negative predictions.

### 3.7. Accuracy with CI 95%

Both models showed high overall accuracy; however, the Scholar AI Free model had a slightly higher point estimate. The overlapping 95% confidence intervals suggested the difference in accuracy between the two models is not statistically significant.

### 3.8. Balanced Accuracy

Scholar AI Free demonstrated a marginally superior performance for moderate and severe classes, achieving perfect balanced accuracy for severe cases. This indicated its superior ability to balance sensitivity and specificity, especially for the severe class.

In conclusion, the Scholar AI Free model appeared to have a slight edge, particularly in the moderate and severe scoliosis classes. Its superior performance in terms of sensitivity, specificity, positive predictive value, negative predictive value, and balanced accuracy for these classes suggested it may be a more reliable tool for scoliosis severity classification. However, the Microsoft Bing model also exhibited strong performance, particularly in the mild class.

#### Comparison of Actual and AI-Predicted Scoliosis Classifications: An Analysis of MAE

In the specific instance of the ChatGPT and Scholar AI Premium classifiers, they achieved perfect classification accuracy for all posturographic images under consideration.

Consequently, the MAE for both these AI systems can be assessed as zero. In contrast, Table 2 provides the evaluation of the MAE for those AI systems whose predictions encompassed misclassifications (i.e., in the case of not perfect classification accuracy). This table furnished a quantitative measure of the average discrepancy between the predicted and actual scoliosis classes for each AI, thereby offering a detailed understanding of the degree and prevalence of misclassification errors in these systems.

For the mild scoliosis class, both models demonstrated perfect classification with an MAE of 0. In the moderate scoliosis class, consisting of 10 cases, the Microsoft Bing model had an MAE of 0.90, while the Scholar AI Free model had a higher MAE of 1.50. This indicated that both models had difficulty accurately classifying the moderate cases, with the Scholar AI Free model exhibiting a greater average deviation (and also misclassifications—see Table 3) from the actual class.

When considering the severe scoliosis class, which comprised 24 cases, the Microsoft Bing model demonstrated an MAE of 1.13, suggesting some misclassifications. However, the Scholar AI Free model perfectly classified all severe cases, as indicated by its MAE of 0.

Overall, for all 56 cases, the Microsoft Bing model yielded an MAE of 0.64, and the Scholar AI Free model achieved a lower MAE of 0.27. Although both models made some misclassifications, the Scholar AI Free model demonstrated a smaller average discrepancy between the predicted and actual scoliosis classes.

In summary, while both models performed flawlessly in classifying mild scoliosis, they struggled to accurately classify the moderate cases. Particularly, the Scholar AI Free model had a larger error in predicting moderate cases but performed impeccably on severe cases, unlike the Microsoft Bing model. Despite these misclassifications, the overall performance of the Scholar AI Free model was better than the Microsoft Bing model, as indicated by its lower overall MAE.

**Figure 2:**
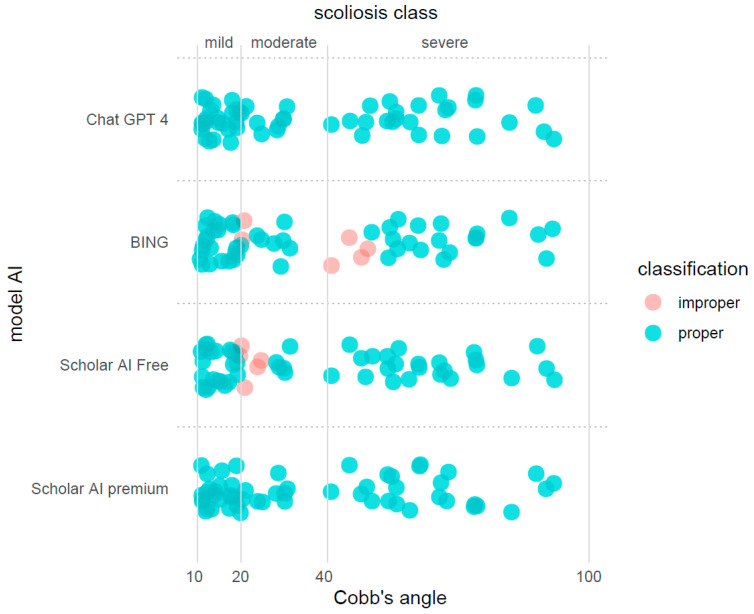
The classification outcomes based on Cobb’s angle measurements illustrated using a jittering procedure to prevent data overlap and enhance visual clarity. Vertical lines were used to demarcate the thresholds that separate the different scoliosis severity classes—mild, moderate, and severe.

An important observation from the analysis is that the majority of the misclassifications occurred for angle measurements that were near the boundary thresholds of the scoliosis severity classes (see Figure 2). Figure 2 highlights any misclassifications, particularly those instances that were positioned near the threshold lines, and provides a more immediate understanding of the AI models’ performance.

Each model, before classifying the scoliosis descriptions, proposed a division of Cobb angles against which it matched the descriptions. The qualifications of the individual models are presented in Table 4.

In Table 4, we observe that only the models ChatGPT 4 and Scholar AI Premium adopted a scoliosis classification partially consistent with independent assessments by specialists in neurosurgery. In contrast, the Scholar AI Free and Microsoft Bing models used different classification criteria, resulting in lower precision in diagnosing the severity of scoliosis. An important aspect to note is that none of the models integrated the complete definition of mild scoliosis based on scientific literature into the classification process. Specifically, the significant threshold for recognizing mild scoliosis, which according to scientific data is defined as a Cobb angle exceeding 10 degrees, was omitted.

In Table 5, we present the arguments provided by the individual AI models during the classification of scoliosis.

## 4. Discussion

The results of our study significantly confirm our hypothesis, demonstrating that among the four tested artificial intelligence models, only two accomplished flawless classification of single-curve scoliosis cases, based on the measurement of Cobb angles.

Our findings indicate that both ChatGPT 4 and Scholar AI Premium achieved impressive accuracy in classifying single-curve scoliosis. Conversely, the Microsoft Bing model exhibited relatively lower accuracy, while Scholar AI Free also demonstrated high effectiveness, yet did not match its more advanced counterpart. This can be attributed to the fact that both Scholar AI Free and Microsoft Bing applied a different classification of scoliosis, resulting in inferior outcomes. Surprisingly, none of the models referred to any scientific data in creating their classification, especially considering that Scholar AI is specifically dedicated to literature research. Moreover, none of the models integrated a complete definition of mild scoliosis based on scientific literature in their classification process. Specifically, an important threshold for recognizing mild scoliosis, identified in scientific data as a Cobb angle exceeding 10 degrees, was overlooked.

The accuracy assessment of artificial intelligence models like ChatGPT and Scholar AI Premium may stem from several key factors that are characteristic of highly advanced AI systems.

### 4.1. Analysis of ChatGPT 4 and Scholar AI Premium Models

Models such as ChatGPT 4, employing advanced machine learning algorithms including deep learning techniques, are capable of demonstrating exceptional precision in identifying subtle patterns in data. ChatGPT, and other general-purpose AI models like GPT 4, are not specifically trained to recognize scoliosis or any other detailed medical tasks [33]. They are trained on a vast and general variety of textual data, allowing them to generate responses based on generally available knowledge and understanding of the context posed by the user [34]. They use this acquired information to attempt to solve specific problems they are directed at by users, but their responses are not a result of dedicated training on specialized data such as radiological descriptions of scoliosis. Instead, their ability to navigate medical topics stems from the general knowledge they have acquired during broad training and is limited to what is generally known and accessible in widely available training data [35,36].

This juxtaposition of complexity and depth in the model, especially evident in the case of ChatGPT 4, which as a fourth-generation model likely possesses a greater number of layers and neurons, enables more effective modeling of complex relationships present in data [37]. In the context of these factors, the high accuracy achieved by ChatGPT 4 and Scholar AI Premium can be viewed as a result of applying the latest advancements in artificial intelligence and machine learning, making these models highly effective in specific medical applications.

### 4.2. Analysis of Scholar AI Free and Microsoft Bing Models

The noticeable difference in the performance of these systems can be attributed to a range of technical and methodological aspects. Let us begin by considering limitations in training data. It is possible that the Microsoft Bing and Scholar AI Free models were trained on datasets that were smaller or exhibited less diversity, which directly impacts the models’ ability to generalize knowledge to new, unknown cases. Furthermore, if these data were noisy or contained errors, it could significantly limit the models’ ability to learn correct patterns. 

Another element is the complexity and architecture of the model. Microsoft Bing and Scholar AI Free might be based on less complex network architectures, which could explain difficulties in modeling more subtle features in the data. The lack of specialization of these models in the specific task of scoliosis identification might also be key to their lower accuracy in this specific application.

The approach to algorithms and learning techniques could also play a significant role. The absence of the latest or most advanced machine learning techniques, such as deep convolutional networks, might be why these models do not match the accuracy of higher-performing ones. Overfitting or underfitting to the training data are additional potential explanations for the observed differences.

### 4.3. Incorrect Classification of Scoliosis by AI Models

In the context of observed inaccuracies in scoliosis classification by all tested AI models, a detailed analysis of potential sources of error seems crucial. The clinical significance of accurate classification cannot be overstated as an erroneous classification of scoliosis could lead to inappropriate clinical decisions. All models committed the same mistake by classifying all cases of spinal curvature as ‘mild scoliosis’, even if the angle was less than 10 degrees, which is inconsistent with medical diagnostic standards. This may indicate serious limitations in the training data—perhaps they were incomplete or incorrectly labeled, leading the models to incorrect conclusions. As highlighted in the article by C. V. von Schacky, the effectiveness of AI in medical diagnostics heavily relies on the quality and comprehensiveness of the training data. The article underscores the importance of using well-curated and diverse datasets, especially in medical applications where the accuracy of diagnosis is paramount [38].

A lack of appropriate diversity in the training data, including an insufficient number of cases with angles below 10 degrees, might have prevented the algorithms from learning that not all detected curvatures qualify as scoliosis. This underscores the necessity of involving medical experts in the process, who could provide valuable insights into diagnostic issues and proper data labeling. Furthermore, the model algorithms did not take into account the specificity of medical criteria for scoliosis diagnosis, failing to appropriately distinguish curvatures below and above the critical 10-degree threshold. This suggests a need for more complex algorithms and verification systems that could better handle medical nuances. As Zezhong Ye discusses in the editorial, the integration of deep learning in medical diagnostics requires not only sophisticated algorithms but also a deep understanding of the medical conditions being analyzed. The editorial emphasizes the importance of algorithmic complexity and the ability to interpret subtle diagnostic criteria, which seems to be lacking in the current AI models for scoliosis classification [39].

The lack of rigorous validation, not encompassing a diverse range of test cases, may also significantly impact the final accuracy of the models. Considering these challenges, it is essential not only to revise and improve training and validation datasets but also to engage in deeper collaboration with medical experts who can provide critical insights into diagnosis. Implementing more complex validation procedures that can mimic real clinical conditions is key to enhancing the effectiveness of AI models in medical applications. Such an approach would ensure not only greater accuracy in diagnosing scoliosis but also increase trust in artificial intelligence systems in medicine.

### 4.4. Lack of References to Scientific Data in AI Models

The issue of AI models not referencing specific scientific studies in their arguments may arise from several reasons.

- Accessibility of Knowledge: AI models might be programmed to utilize publicly available and widely recognized information, often accepted as standard or clinical practice. If the classification is based on broadly accepted practices, the model may not need to refer to specific studies if this information is encoded in its knowledge base as generally accepted. However, a study on AI hallucination in scientific writing through ChatGPT references found that a significant number of references listed by ChatGPT did not have a Digital Object Identifier (DOI) or were not found in Google searches, suggesting limitations in ChatGPT’s ability to generate reliable references [40].
- Data Processing: AI models might use advanced natural language processing techniques, enabling them to understand and apply knowledge from various sources. Although they have access to vast databases, their ability to cite specific sources in real-time may be limited by their programming or algorithms, prioritizing easily accessible information and response speed over the depth and detail of scientific references. This is exemplified in the phenomenon of hallucination in large language models, where they occasionally produce outputs that deviate from user input or factual knowledge [41].
- Interface and Usage Limitations: Some platforms using AI may restrict the length or complexity of responses, consequently limiting the model’s ability to present detailed arguments based on specific scientific research. This limitation can contribute to the issue of hallucination, where AI models generate content that diverges from the real facts, resulting in unfaithful outputs [42].
- Interpretation and Flexibility: Medical knowledge is often interpreted and modeled by AI algorithms in a flexible manner to fit many different contexts. This could result in a preference for more general statements instead of specific references to studies, especially when there are multiple studies with varying outcomes or interpretations. The challenge is further compounded by the nuanced categorization of hallucination in AI models, which can manifest in various forms and degrees of severity, affecting the reliability and specificity of the information provided [42].

In summary, AI models may be optimized for delivering quick and practice-consistent responses but may not be tailored to provide detailed scientific references in every response, especially if it is not directly required for understanding or responding to a query.

Currently, there is an increasing number of scientific studies focusing on exploring the capabilities of advanced artificial intelligence systems, such as ChatGPT, Microsoft Bing, or Scholar AI, in the context of diagnostic support. 

The studies by Kumari et al., A. Arjomandi et al., and others explore the application of LLMs and AI in healthcare. Kumari et al. focus on the performance of ChatGPT 3.5, Google Bard, and Microsoft Bing in hematology, finding ChatGPT to be the most efficient, suggesting the need for further model development for clinical use [43]. Arjomandi et al. examine AI and natural language processing (NLP) in cardiothoracic surgery, highlighting their role in advancing diagnosis, treatment, and healthcare personalization through data analysis [44]. Another study emphasizes ChatGPT’s contribution to Clinical Decision Support Systems (CDSS), demonstrating its capacity to provide real-time, evidence-based recommendations for medical professionals, thereby enhancing healthcare efficiency [45].

Our research project encountered several limitations that could have influenced the results and interpretations. Since the AI models were not specifically trained for this study, their performance might have been limited by the data on which they were originally trained. We did not have control over the composition of the training data nor influence over the process of their selection, which could limit the models’ ability to generalize the results to other, non-specific data sets. Uncertainty about the quality and scope of the training data used by the model creators could affect the accuracy and reliability of AI in our specific application. To address these limitations, long-term studies with a larger and more diverse patient sample are recommended, encompassing various types of scoliosis and a broader age range. For a better understanding of the potential and limitations of individual AI models, gaining more knowledge about the training data used by manufacturers, as well as conducting our training and optimization process of the models if possible, is also advisable. Collaboration with medical experts and radiologists could also contribute to a better understanding and interpretation of the results, as well as to the creation of more detailed and specific training and validation data sets, which would be more representative of real clinical conditions.

## 5. Conclusions

The AI models ChatGPT 4 and Scholar AI Premium demonstrated significant accuracy in identifying single-curve scoliosis, highlighting their potential in medical diagnostics and the importance of advanced machine learning technologies. Despite the lack of specialized medical training, they achieved high efficiency, indicating the capabilities of general AI models trained on broad data sets. Meanwhile, other models, such as Microsoft Bing and Scholar AI Free, showed lower accuracy, likely due to limitations in their training data and architecture. However, all models made the same mistake in classifying cases of mild scoliosis, suggesting a need for improvement in training data and the necessity of involving medical experts in the AI training process to ensure realistic labeling. It was also emphasized that complex validation procedures, which would mirror real clinical cases, are crucial for increasing AI’s credibility in medicine. The study results indicate the enormous potential of AI in analyzing large sets of medical data, which could revolutionize diagnostics and the personalization of healthcare. Moreover, the diversity of AI models’ competencies indicates the need for their further development to more effectively meet specific needs in clinical practice.

## Figures and Tables

**Table 1:** The results of interrater agreement and performance evaluation metrics between actual and predicted classes of scoliosis.

Parameter	Class	n	AI Model			
			ChatGPT 4	Microsoft Bing	Scholar AI Free	Scholar AI Premium
Fleiss’ Kappa *	mild	22	1.00	0.93	0.82	1.00
	moderate	10	1.00	0.66	0.62	1.00
	severe	24	1.00	0.85	1.00	1.00
	Overall	56	1.00	0.83	0.85	1.00
Sensitivity	mild	22	1.00	0.92	0.81	1.00
	moderate	10	1.00	0.67	1.00	1.00
	severe	24	1.00	1.00	1.00	1.00
Specificity	mild	22	1.00	1.00	1.00	1.00
	moderate	10	1.00	0.95	0.90	1.00
	severe	24	1.00	0.89	1.00	1.00
Positive predictive value	mild	22	1.00	1.00	1.00	1.00
	moderate	10	1.00	0.80	0.50	1.00
	severe	24	1.00	0.83	1.00	1.00
Negative predictive value	mild	22	1.00	0.94	0.85	1.00
	moderate	10	1.00	0.91	1.00	1.00
	severe	24	1.00	1.00	1.00	1.00
Accuracy with CI 95%	Overall	56	1.00 [0.94, 1.00]	0.89 [0.78, 0.96]	0.91 [0.80, 0.97]	1.00 [0.94, 1.00]
Balanced accuracy	mild	22	1.00	0.96	0.91	1.00
	moderate	10	1.00	0.81	0.95	1.00
	severe	24	1.00	0.94	1.00	1.00

Note: * all indices were significant *p* < 0.001.

**Table 2:** The MAE estimates for AI with one class of scoliosis misclassification.

Class	N	AI Model	
		Microsoft Bing	Scholar AI Free
Mild	22	0	0
Moderate	10	0.90	1.50
Severe	24	1.13	0
Overall	56	0.64	0.27

**Table 3:** Confusion matrix for studied AIs.

AI Model	Actual Class	Predicted Class		
		Mild	Moderate	Severe
Chat GPT 4, Bing, Scholar free, Scholar premium	Mild	22	0	0
Chat GPT 4, Scholar premium	Moderate	0	10	0
Bing		2	8	0
Scholar free		5	5	0
Chat GPT 4, Scholar free, Scholar premium	Severe	0	0	24
Bing		0	4	20

**Table 4:** The division of scoliosis according to Cobb angle as proposed by the AI model, based on which it classified the implemented descriptions.

AI Model	Proposed Classification of Scoliosis
ChatGPT 4	Mild scoliosis: Cobb angle less than 20 degrees. Moderate scoliosis: Cobb angle between 20 and 40 degrees. Severe scoliosis: Cobb angle greater than 40 degrees.
Microsoft Bing	Mild scoliosis: Cobb angle less than 20 degrees. Moderate scoliosis: Cobb angle between 20 and 50 degrees. Severe scoliosis: Cobb angle greater than 50 degrees.
Scholar AI Free	Mild scoliosis: Cobb angle less than 25 degrees. Moderate scoliosis: Cobb angle between 25 and 40 degrees. Severe scoliosis: Cobb angle greater than 40 degrees.
Scholar AI Premium	Mild scoliosis: Cobb angle less than 20 degrees. Moderate scoliosis: Cobb angle between 20 and 40 degrees. Severe scoliosis: Cobb angle greater than 40 degrees.

**Table 5:** The AI models’ rationale regarding reference to source data.

AI Model	Rationale
ChatGPT 4	‘The classification I used for assessing scoliosis based on the Cobb angle is widely accepted and used in clinical practice.’
Microsoft Bing	‘The division I presented earlier was somewhat simplified. A more detailed division, which I found, is consistent with information provided on the Spine-health website [https://www.spine-health.com/conditions/scoliosis/cobb-angle-measurement-and-treatment-guidelines (accessed on 29 October 2023)].’
Scholar AI Free	‘The classification of scoliosis based on Cobb angles that I presented is a generally accepted practice in medicine. However, it is not information that I derived from a specific scientific source.’
Scholar AI Premium	‘I am sorry, but unfortunately, I could not find a specific scientific study that precisely defines the ranges for mild, moderate, and severe scoliosis based on the Cobb angle. All the articles I found pertain to different aspects of scoliosis and its measurement, but none of them directly answers your question. Nonetheless, the classification I used for assessing scoliosis based on the Cobb angle is widely accepted and used in clinical practice.’

## Data Availability

Data are contained within the article.
